# Enhancing genomic association studies in slash pine through close-range UAV-based morphological phenotyping

**DOI:** 10.48130/forres-0024-0022

**Published:** 2024-07-25

**Authors:** Ruiye Yan, Yihan Dong, Yanjie Li, Cong Xu, Qifu Luan, Shu Diao, Chunyan Wu

**Affiliations:** 1 State Key Laboratory of Tree Genetics and Breeding, Research Institute of Subtropical Forestry, Chinese Academy of Forestry, Hangzhou 311400, Zhejiang, China; 2 College of Landscape and Travel, Agricultural University of Hebei, Baoding 071051, Hebei, China; 3 School of Forestry, University of Canterbury, Private Bag 4800, Christchurch 8140, New Zealand; 4 State Key Laboratory of Tree Genetics and Breeding, Key Laboratory of Tree Breeding and Cultivation of National Forestry and Grassland Administration, Research Institute of Forestry, Chinese Academy of Forestry, Beijing 100091, China

**Keywords:** UAV, Morphological traits, Genome-wide association studies (GWAS), Tree phenotyping, Genetic variations

## Abstract

In forestry genetics and industry, tree morphological traits such as height, crown size, and shape are critical for understanding growth dynamics and productivity. Traditional methods for measuring these traits are limited in efficiency, scalability, and accuracy, posing challenges for large-scale forest assessments. This study focuses on integrating unmanned aerial vehicle (UAV) technology with GWAS to improve genomic association studies in slash pine (*Pinus elliottii*). Seven key morphological traits have been identified (canopy area (CA), crown base height (CBH), crown length (CL), canopy volume (CV), crown width (CW), crown width height (CWH), and tree height (H)) through advanced UAV-based phenotyping. These associations account for a remarkable range of heritability in slash pine, with traits such as CBH, CL, CV, and H showing relatively high heritability across both Single nucleotide polymorphisms (SNP) and pedigree methods, indicating strong genetic influence, while traits such as CWH show lower heritability, suggesting greater environmental influence or non-additive genetic variance. The GWAS identified 28 associations, including 22 different SNPs localized to 16 candidate genes, that were significantly associated with the morphological traits of Slash Pine. Notably, two of these candidate genes, annotated as putative DEAD-like helicase and ethylene-responsive element binding factor (ERF), were present at different mutation sites and were significantly associated with CW and CA traits, respectively. These results demonstrate that the UAV imaging enables a comprehensive analysis of the Morphological growth response of slash pine and can facilitate the discovery of informative alleles to elucidate the genetic structure underlying complex phenotypic variation in conifers.

## Introduction

The morphological phenotypes of trees, including tree height, crown spread, crown length, crown width, height to the lowest branch, crown volume, and height at the widest part of the crown, are fundamental to understanding ecological and environmental dynamics^[1,2]^. These phenotypic attributes are critical not only as indicators of a tree's health and growth patterns but also as integral components in the broader context of global ecological balance. The study of these morphological traits extends beyond simple physical measurements, significantly contributing to our understanding of ecosystem services, biodiversity, and the impact of environmental changes on forest health^[3]^.

Digital Whole-Community Phenotyping (DWCP) exemplifies advancements in capturing these essential phenotypic data by collecting high-resolution multispectral and structural data on plant communities^[4]^. This novel approach underscores the importance of precise phenotypic data collection in advancing ecological research.

In the field of forestry genetics and tree breeding, the detailed analysis of tree morphological phenotypes is paramount. Tree architecture and functional traits are closely interlinked, providing insights into tree life history and demography, essential for genetic studies and breeding programs^[5−7]^. These characteristics directly influence the timber industry, impacting wood quality, yield, and overall forest productivity. Rapid advancements in sensor technologies and image-based phenotyping are transforming how these traits are measured and analyzed, offering new avenues for accelerating forest breeding^[8]^. Accurate measurement and analysis of these phenotypic traits are crucial for advancing genetic selection and breeding programs aimed at enhancing timber production and forest sustainability. Ongoing research and development in forest tree functional genomics and breeding highlight the critical role of these traits in meeting the growing global demand for wood fiber and other bioproducts from forest trees^[9]^. The development and improvement of these traits through selective breeding and genetic studies hold immense potential for the future of the timber industry and ecological conservation efforts globally^[10]^.

In forestry research, traditional methods for assessing tree morphological phenotypes have primarily relied on manual and direct observational techniques. These approaches involved measuring various physical attributes, including tree height, crown dimensions, and other relevant phenotypic traits. While these methods have historically provided foundational insights, they have notable limitations in efficiency and scalability^[8,11,12]^. Manual measurements are labor-intensive and subjective, often resulting in significant time investments and potential inconsistencies in data accuracy. These limitations are particularly pronounced when dealing with extensive datasets or conducting wide-area forest surveys, where rapid and accurate data collection is essential^[13]^. Additionally, the low-throughput nature of traditional methods poses a significant challenge in large-scale ecological studies and comprehensive forest monitoring efforts. The difficulty in applying these techniques in varied and often inaccessible terrains further restricts their utility across diverse ecological landscapes^[14]^.

The demands of contemporary forestry research, especially in contexts requiring prompt and precise data gathering—such as monitoring dynamic environmental impacts on forest health or managing extensive commercial forestry operations—necessitate the evolution of phenotyping methodologies^[15,16]^. This need has driven the exploration and adoption of more sophisticated, efficient, and accurate phenotypic data collection techniques, paving the way for innovative approaches in forestry and ecological studies^[17]^.

The advent of Unmanned Aerial Vehicle (UAV) technology and Structure from Motion (SFM) imaging methods has revolutionized forestry phenotyping. SFM, a photogrammetric technique that uses 2D images to reconstruct 3D structures, has emerged as a powerful tool in forestry research. By capturing multiple overlapping images of a forested area from different angles, SFM processes these images to create a 3D model or point cloud of the area. This method is particularly advantageous for providing high-resolution data on forest canopy structure and tree morphology, which are essential for assessing biomass, forest health, and growth patterns^[18]^.

The superiority of SFM methods lies not only in their accuracy and detail but also in their ability to cover large areas rapidly and frequently, allowing for dynamic monitoring of forests^[19]^. This technology offers a non-invasive, cost-effective, and highly scalable approach to forest monitoring, making it invaluable for modern forestry management, ecological research, and conservation efforts. By facilitating detailed and regular assessments of forest structures, UAV technology helps in understanding forest dynamics, carbon sequestration potential, and the impact of environmental changes, playing a crucial role in sustainable forest management and climate change studies^[20]^.

Genome-Wide Association Studies (GWAS) have become increasingly vital in forest genetics, particularly for identifying key genes associated with morphological phenotypic traits^[21]^. This molecular breeding approach holds significant promise for enhancing our understanding of the genetic basis of critical traits like tree height, crown architecture, and overall growth patterns. Identifying these key genes is crucial for advancing forestry genetics and breeding programs, as it enables the development of tree varieties with improved timber quality, disease resistance, and adaptability to environmental changes^[22−24]^.

Despite the popularity of UAV technology in phenotyping and the significance of GWAS in molecular breeding, the integration of high-throughput phenotyping with genotyping remains underexplored, especially in species like slash pine (*Pinus elliottii*)^[25]^. The challenges are compounded by the fact that slash pine, a large coniferous tree, and a gymnosperm, has not yet had its genome fully sequenced. The complexity of its genome, typical of many large woody perennials, adds another layer of difficulty. While UAV and GWAS individually contribute significantly to the field, their combined application in the genetic improvement and selection of key candidate genes in species like slash pine represent a burgeoning field of study with immense potential yet to be fully explored^[26,27]^.

In response to these challenges, the present research group is pioneering the resequencing of slash pine. Utilizing genomic data from closely related sequenced species and combining it with transcriptome sequencing, a 51k liquid-phased probe array has been developed and specifically designed for slash pine^[28]^. This tool enables targeted resequencing to capture the genetic variations most pertinent to slash pine's morphological traits. This approach significantly enhances our understanding of slash pine's genetic architecture and paves the way for future breeding programs. By linking phenotypic traits with their genetic underpinnings, the aim is to improve the growth rate and wood quality in slash pine.

Therefore, in this study, we used a slash pine plantation to achieve two primary objectives:

1) Employ UAV imaging for efficient, high-throughput quantification of slash pine morphological phenotypes. The aim is to systematically collect detailed data on vital traits (canopy area (CA), crown base height (CBH), crown length (CL), canopy volume (CV), crown width (CW), crown width height (CWH) and tree height (H)), which are essential for deciphering growth patterns and structural variations in slash pine.

2) Integrate phenotypic data derived from UAV technology with genomic resequencing data to perform a targeted GWAS. This phase focuses on identifying candidate genes that show a significant correlation with the morphological attributes of slash pine, thereby providing valuable insights into the genetic determinants of these traits within the constraints of our sample size.

By achieving these objectives, this study addresses a critical gap in current research by combining advanced UAV phenotyping with genomic analyses, providing comprehensive insights into the genetic and phenotypic variations in slash pine.

## Materials and methods

### Site description

This study was conducted across two meticulously managed slash pine (*Pinus elliottii*) plantations within the Matou National Forest Farm, located in Jingxian County, Xuancheng City, Anhui Province, China. The first site is positioned precisely at the coordinates of 30°45'N and 118°29'E, while the specifics of the second site's location remain closely aligned within the same geographic and climatic zone, ensuring comparable environmental conditions. Both sites are typified by a temperate climate, exhibiting an average annual temperature of 15.7 °C. The thermal profile spans from the chilliest month, January, averaging 2.9 °C, to the peak of warmth in July, averaging 28.1 °C. Precipitation across these regions averages 1,525 mm annually, coupled with an average relative humidity of 84%. The soil in these plantations predominantly consists of yellow loam, characterized by an acidic to neutral pH of 5.5 to 6.0 and depths ranging from 70 to 150 cm. Established in 2013, each plantation spans three hectares, collectively covering six hectares, and is composed of 20 genetically diverse open-pollinated families, each tracing back to the same maternal progenitor. The plantation's design is a lattice incomplete block with single tree plots, where each block, measuring 20 trees with a spacing of 6 m × 8 m, represents an individual family without replication within the block. The entire experimental area across both sites comprises 40 such blocks, effectively splitting the previously singular experimental setup into two distinct but similar environments for comparative analysis. Detailed characteristics and historical data of these plantations have been documented in preceding publications by the same research team^[29−31]^.

### UAV RGB images acquisition

The UAV imagery was acquired using a DJI Matrice 300 RTK (M300RTK) drone, which was equipped with a P1 35 mm camera, provided by Dà-Jiāng Innovations Science and Technology Co., Ltd., China. The flight operation was conducted on December 24, 2023, under optimal weather conditions characterized by clear skies and minimal wind. The M300RTK drone, renowned for its stability and precision, was remotely controlled to maintain a consistent flight altitude, ensuring uniform image quality and coverage.

The camera, designed for advanced aerial photography, employed a swing shooting mode to capture detailed images of the plantation. We meticulously planned the flight path to mirror a double grid pattern, similar to our previous methodology, ensuring 80% image overlap. This strategic approach was crucial for achieving comprehensive coverage of the entire plantation area in a single flight. The M300RTK's advanced flight capabilities allowed us to maintain a forward speed of 8 m/s, optimizing the efficiency of the image capture process.

One of the key features of the M300RTK drone is its integration with a networked real-time kinematic (RTK) positioning system. This system provided highly accurate waypoint positioning, significantly reducing horizontal and vertical positioning errors to 0.03 m and 0.06 m, respectively. The precision of this technology was instrumental in ensuring the high quality of the spatial data acquired during the flight, which lasted approximately 40 min.

To ensure the reproducibility and robustness of the data, multiple flights were conducted under similar conditions, maintaining the same flight altitude of 40 m and clear weather for each session. The use of RTK precise positioning across all flights ensured consistency and accuracy in georeferencing. These repeated flights validated the reproducibility of the image acquisition process, as evidenced by the consistency of the phenotypic data. Previous studies have further confirmed the reliability and robustness of the UAV-based phenotyping approach^[29,30]^.

### Image processing

Utilizing high-precision RGB imagery, the data processing employed the DJI Terra software (version 3.3.0, Shenzhen, China) for generating orthoimages and 3D point cloud data. In this study, the algorithm originally proposed by Song et al. was refined and enhanced, focusing on increased accuracy and speed in data extraction^[29]^. The approach minimized manual intervention by employing automated processes, significantly improving the efficiency of handling large data volumes. Previous studies have validated the accuracy of these methods using field data such as tree height and diameter at breast height (DBH)^[29−31]^. These validations demonstrated that the UAV-based measurements are highly reliable and consistent with traditional ground-truth measurements.

The lidR package (version 4.0.0) and the terra package (version 1.7-39) in R (R Core Team, 2023) were instrumental in our individual tree analysis and phenotypic data extraction. The workflow commenced with the generation of Digital Terrain Models (DTMs) and Digital Surface Models (DSMs) from the 3D point cloud data. The Cloth Simulation Filtering (CSF) algorithm^[32]^ effectively differentiated ground points to produce DTMs, while DSMs were constructed using a point-to-raster algorithm. Subsequently, the Canopy Height Model (CHM) was derived from the differential between DTM and DSM.

For tree detection and segmentation within the plantation, the dalponte2016 function from the lidR package was used. This function was configured to detect trees with a minimum height of 2.6 m and a maximum crown radius of 2.5 m. Subsequently, individual tree point clouds were extracted, with a focus on removing noise and irrelevant data points to ensure the purity of the dataset.

Following this initial processing of the point cloud, the attention turned to the in-depth analysis of individual tree data to accurately determine morphological traits. This intricate process was facilitated by a series of custom-developed R functions, designed to handle specific aspects of the point cloud data. The following steps delineate the methodology:

The calculate_CBH_LAS function estimated Crown Base Height by identifying the branching point above the trunk, using a combination of height thresholds and standard deviation calculations.

calculate_canopy_height_outside_trunk assessed the canopy height outside the trunk's radius, providing a comprehensive view of the tree's overall height.

The calculate_crown_width function evaluated the crown's maximum width by calculating the greatest horizontal distance across the canopy.

calculate_extreme_points_height determined the maximum height reached by the canopy, offering insights into the vertical extent of the tree's growth.

Finally, calculate_crown_volume_ellipsoid computed the crown volume, applying an ellipsoidal model to the canopy dimensions derived from the point cloud.

These functions, fine-tuned for slash pine characteristics, were applied to the individual tree point cloud data. By processing the point cloud through these custom R functions, detailed morphological measurements for each tree were extracted. This included data on tree height, crown base height, crown width, the height at the widest part of the canopy, and overall crown volume. By integrating these measurements, a comprehensive phenotypic profile for each tree was ensured, providing a detailed dataset for subsequent GWAS studies.

### SNP probe

The genotyping aspect of the present study was underpinned by the creation of a novel 51k SNP probe array for slash pine, devised using the genotyping by target sequencing (GBTS) method based on solution hybridization. This development was informed by a comprehensive collection of SNP data and EST-probe sequences from related pine species, as well as the present research groups own extensive slash pine specimen and transcriptome data. Specifically, the following were incorporated:

1. A combination of SNP loci from loblolly pine (*Pinus taeda*), fast-growing slash pine specimens, and transcriptomes of open-pollinated slash pine offspring.

2. EST-probe sequences from loblolly pine.

3. SNPs associated with various traits in loblolly pine.

These components were meticulously mapped against the loblolly pine reference genome, resulting in a robust SNP array encompassing over 51,000 probes. In December 2022, a targeted collection of 1-year-old needle samples from 200 trees, spanning 20 families, across four canopy directions was conducted. These samples were immediately preserved and processed for targeted sequencing using the custom SNP array. The initial dataset comprised hundreds of thousands of SNP loci. After stringent filtering for biallelic genes, minor allele frequency (MAF), and call rate criteria, this dataset was refined to approximately 51,630 high-quality SNP loci. These loci formed the basis of our GWAS analysis, providing a critical genetic insight into the studied population. Details on the development and validation of the 51k SNP probe array are provided in a recently published article^[28]^, which thoroughly describes the SNP array design pipeline, the number of high-quality validated SNPs, and the distribution of SNPs across various functional groups and chromosomes.

### Genetic variation and correlations for morphological traits

In this study, three types of data were integrated: individual morphological phenotypes (CA, CBH, CL, CV, CW, CWH, and H), pedigree information, and SNP data. This combination provided the input data for estimating the genetic parameters of growth traits in slash pine. A Generalized Linear Mixed Model (GLMM) was applied, using the restricted maximum likelihood (REML) method *via* the Sommer package in R software^[33]^. In the GLMM:

1 $ y=\left[\begin{array}{c}{y}_{1}\\ {y}_{2}\\ {y}_{i}\end{array}\right]=Xm+{Z}_{1}b+{Z}_{2}f+e $

*y* represents the vector of observed growth traits (crown area, tree height, etc.). $ Xm $ captures the fixed effects, including environmental and treatment effects. $ {Z}_{1}b $ and $ {Z}_{2}f $ represent the random effects matrices for block and family effects, respectively. *b* is the vector of random block effects. *f* is the vector of random family effects. *e* denotes the residual effects.

To estimate the narrow-sense heritability (*h*^*2*^) and genetic correlations ($ {r}_{{g}_{ij}} $) between traits, both pedigree-based and SNP-based methods were utilized. The pedigree-based method inferred heritability based on familial relationships, and the SNP-based method provided a more precise estimation by using individual genetic variants.

The equations for calculating *h*^*2*^ and $ {r}_{{g}_{ij}} $using the pedigree-based method were:

2 $ {h}_{i}^{2}=\dfrac{2.5{\sigma }_{fi}^{2}}{{\sigma }_{fi}^{2}+{\sigma }_{bi}^{2}+{\sigma }_{ei}^{2}} $

where $ {\sigma }_{fi}^{2} $, $ {\sigma }_{bi}^{2} $, and $ {\sigma }_{ei}^{2} $represent the variances due to family, block, and residual effects, respectively.

3 $ {r}_{{g}_{ij}=}\dfrac{{\sigma }_{  {f}_{ij}}^{}}{\sqrt{{\sigma }_{{f}_{i}}^{2}+{\sigma }_{{f}_{j}}^{2}}} $

where $ {\sigma }_{  {f}_{ij}}^{} $ is the covariance between traits *i* and *j* due to family effects.

For the SNP-based method:

4 $ {h}_{SNP}^{2}=\dfrac{2.5{\sigma }_{g}^{2}}{{\sigma }_{g}^{2}+{\sigma }_{e}^{2}} $

where $ {\sigma }_{g}^{2} $ and $ {\sigma }_{e}^{2} $ are the variances due to genotype and environmental effects, respectively.

5 $ {r}_{{g}_{ij}}=\dfrac{\sum \left({\mathrm{\beta }}_{i1}{\mathrm{\beta }}_{i2}\right)/\mathrm{m}}{\sqrt{\sum \left(\dfrac{{\beta }_{i1}^{2}}{m}\right)\sum \left(\dfrac{{\beta }_{i2}^{2}}{m}\right)}} $

with $ {\mathrm{\beta }}_{i1} $ and $ {\mathrm{\beta }}_{i2} $ being the effect sizes of the i-th SNP on traits 1 and 2, and *m* representing the total number of SNPs. The Breeding Value (BV) was calculated as BV = G + G × E, where, G represents the Genetic Effect, encompassing additive genetic effects, and G × E indicates the gene-by-environment interaction.

Analysis and visualization of these genetic parameters, particularly in relation to the morphological traits of slash pine, were conducted using the ggplot2 package^[34]^ in R. This methodology enabled a detailed understanding of the genetic influences on the growth and development of slash pine.

### GWAS for morphological traits

In this study, the statgenGWAS package^[35]^ was utilized for GWAS analysis, focusing on the association between individual SNPs and specific morphological traits of slash pine, namely crown area and tree height. Each SNP locus was independently assessed through a generalized least squares (GLS) model, where the correlations with the morphological traits were determined. This approach was critical for revealing genetic factors influencing the growth characteristics of slash pine.

A kinship matrix, calculated using the 'Yang' method from the Genome-wide Complex Trait Analysis software^[36]^, was incorporated as a covariate in the model to account for fine-scale genetic differentiation. This inclusion allowed for a comprehensive analysis of genetic relationships and variations among the slash pine samples.

The GWAS results were visualized using the CMplot R package^[37]^. The kinship heatmap was illustrated with the ggplot2 package. Detection thresholds were established at 4 and 5 for significance levels of α < 0.05 and α < 0.01, respectively. SNP annotation was conducted using snpEff sv4.5^[38]^. This process included generating a Generic Feature Format (GFF) file of slash pine probe sequences, constructing a specific snpEff database for slash pine, and annotating the SNP array for the 210 analyzed slash pine samples.

## Results

**Figure 1:**
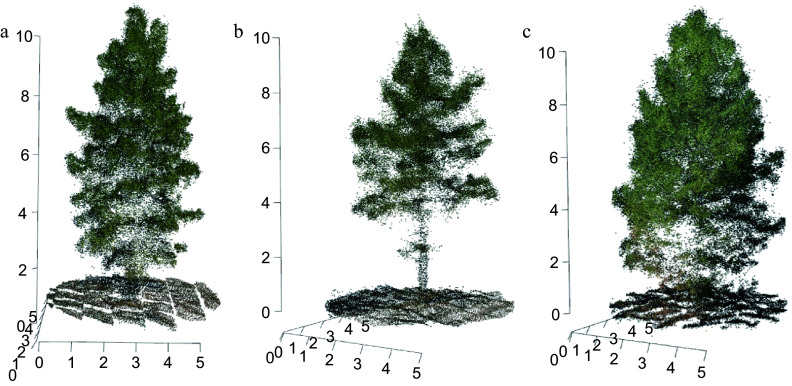
Three-dimensional point cloud visualizations of slash pine trees derived from UAV imaging. (a) Tree with a lower canopy structure, (b) elevated canopy structure, and (c) lower crown with understory.

The UAV imaging provided detailed three-dimensional point clouds of slash pine trees, capturing significant variations in canopy architecture and trunk visibility, as illustrated in Fig. 1. The figure shows three distinct structural forms of the trees. In Fig. 1a, the tree features a lower canopy structure, allowing for clear visibility of the trunk. This structural form can be critical for studies focused on trunk accessibility and lower canopy dynamics. Figure 1b depicts a tree with an elevated canopy, characterized by a pronounced lower crown and visible understory. The separation between the crown base and the trunk is more distinct in this structure, which can be useful for analyzing crown-base height and understory interactions. Figure 1c shows a tree with a notably low crown base, resulting in minimal trunk visibility and a denser understory. This configuration might affect light penetration and understory growth, making it relevant for ecological stratification studies. These phenotypic variations among the trees highlight the diversity within the slash pine plantation. Such diversity is pertinent to understanding adaptive traits, photosynthetic efficiency, and the overall ecological dynamics of the forest canopy. The precise UAV imagery facilitates detailed analysis of structural complexity and functional dynamics in forest environments. This tripartite representation underscores the phenotypic diversity observed within this slash pine plantation, offering insights into the structural complexity and functional dynamics of forest canopies.

**Figure 2:**
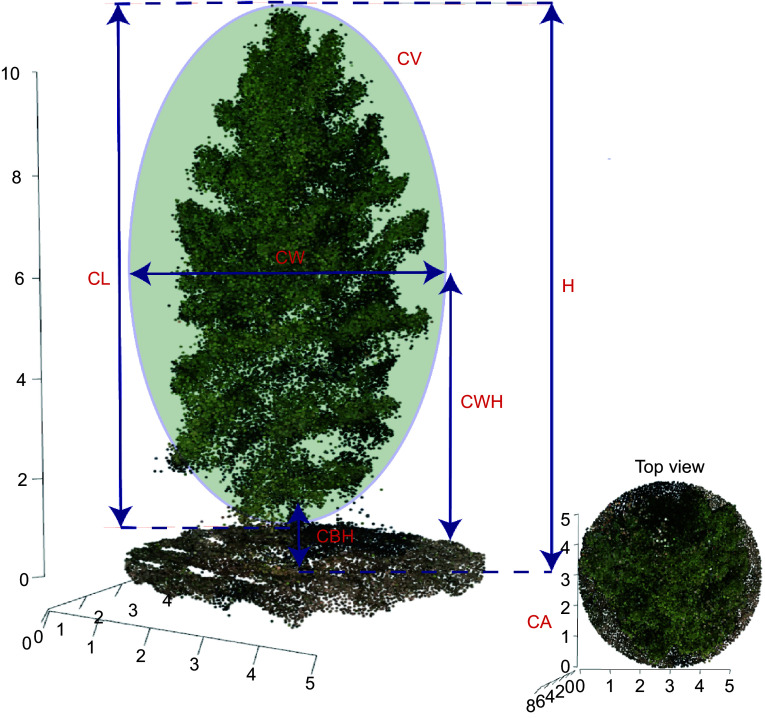
Diagrammatic representation of morphological trait measurement methods in slash pine using UAV-derived 3D point blouds. CA: canopy area, CBH: crown base height, CL:crown length, CV: canopy volume, CW:crown width, CWH: crown width height, H: tree height.

Figure 2 provides a detailed illustration of the methodologies used to measure morphological traits in slash pine, utilizing three-dimensional point cloud data acquired from UAV imagery. This figure delineates key morphological features such as canopy area (CA), crown base height (CBH), crown length (CL), canopy volume (CV), crown width (CW), crown width height (CWH), and tree height (H). The top view inset offers a comprehensive perspective of the crown area, allowing for accurate measurement of the canopy's spatial dimensions. These measurements are critical for understanding the physical structure and potential growth patterns of the trees. To evaluate the accuracy of the present model, correlation analysis was conducted between the measured and predicted values of these morphological traits. The results, shown in Supplemental Fig. S1, illustrate the relationship between the actual and predicted values through scatter plots and calculate the coefficient of determination (R^2^). The R^2^ values for each morphological trait were all above 0.9, indicating the high predictive accuracy of the present model. This high level of accuracy demonstrates the reliability of using UAV-derived 3D point clouds for detailed phenotypic analysis in forestry research.

**Figure 3:**
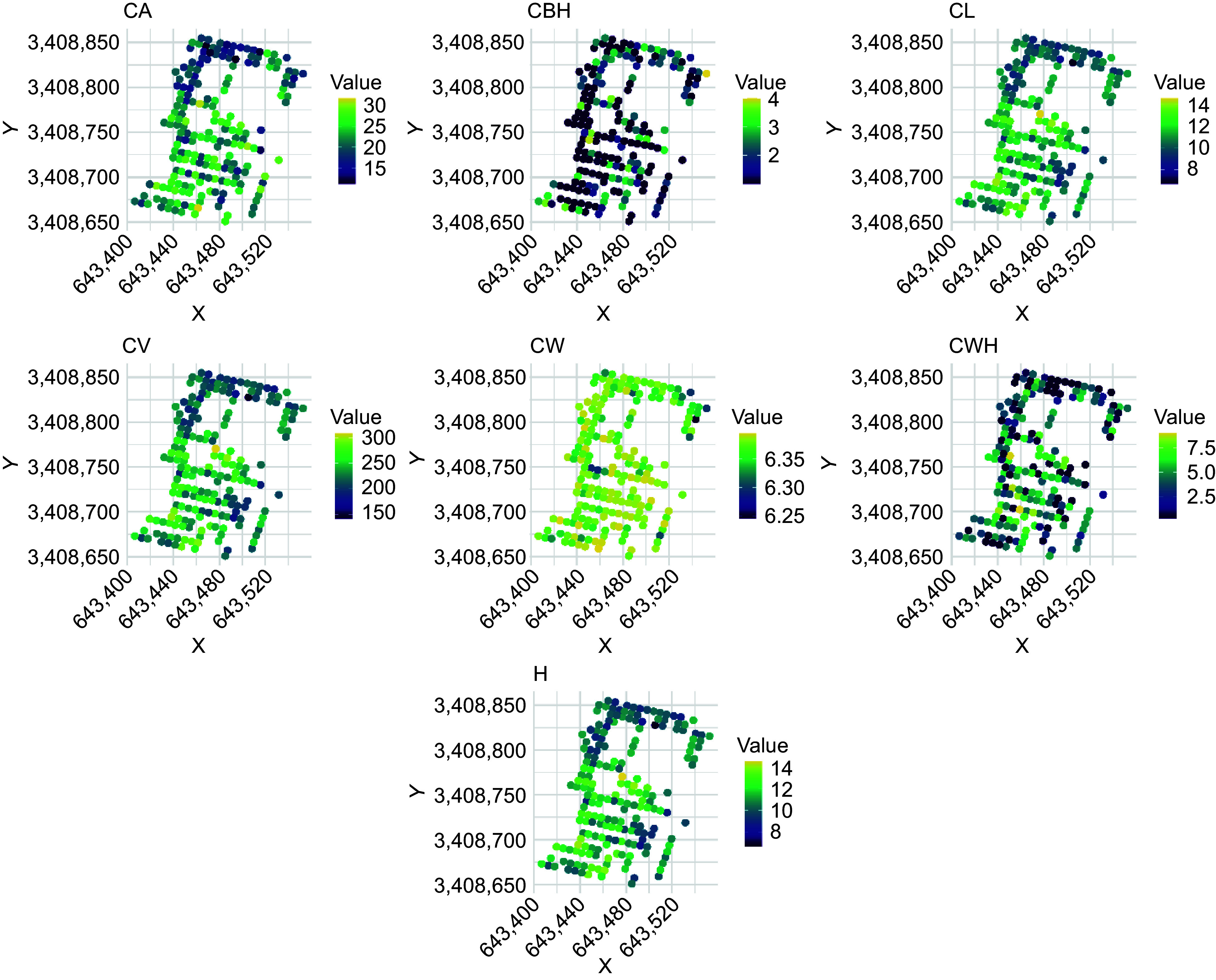
Spatial distribution of morphological traits in slash pine assessed by UAV-derived phenotyping. CA: canopy area, CBH: crown base height, CL: crown length, CV: canopy volume, CW: crown width, CWH: crown width height, H: tree height.

Figure 3 illustrates the spatial distribution of various morphological traits in slash pine as assessed by UAV-derived phenotyping. The collection of scatterplots shows individual phenotypic traits of the trees, with each subplot representing a different trait: CA, CBH, CL, CV, CW, CWH, H. CW values are predominantly high across the sampled population, suggesting either trait-wide robustness or environmental conditions that favor wide crowns. Conversely, the majority of CBH measurements are significantly low, indicating a commonality in lower crown initiation across trees. Both CL and H show similar ranges in value distribution, suggesting a correlative growth pattern between vertical crown extent and total tree height. These spatial distributions provide valuable insight into the phenotypic variability and potential environmental influences on morphological traits of lodgepole pine. Mapping these traits to geographic coordinates provides a comprehensive understanding of their growth dynamics and structural diversity across the sampled terrain.

**Figure 4:**
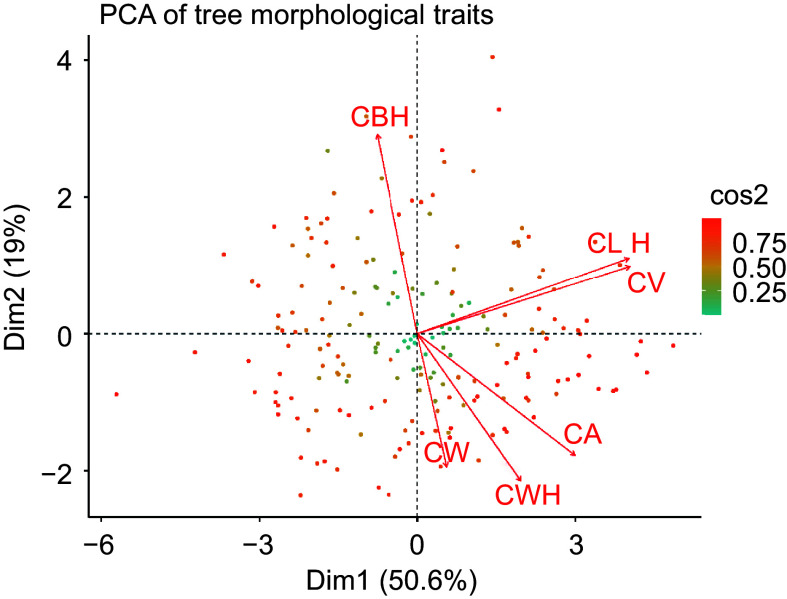
Principal component analysis of morphological trait covariation in slash pine. Each point represents an individual tree, color-coded according to the cos2 value, which indicates the quality of representation of the tree in the PCA plot. Each arrow indicates the direction and strength of the correlation between the respective morphological trait and the principal components.

Principal component analysis (PCA), shown in Fig. 4, elucidates the underlying structure of variability in morphological traits within a slash pine population. This analysis distills a multidimensional data set into principal components, highlighting the primary axes of variance and covariation among key traits. Dimension 1 (Dim1) encapsulates 50.6% of the trait variability, suggesting a strong gradient of correlated morphological features within the dataset. Dimension 2 (Dim2) accounts for an additional 19%, capturing additional variability related to specific morphological traits. Vector orientations in the biplot, depicted as arrows, manifests the loadings of individual traits on the principal components. The proximity and orientation of the vectors corresponding to crown width (CW), canopy area (CA), and crown width height (CWH) suggest a notable positive correlation among these traits. Similarly, the orientation of the vectors for crown length (CL), canopy volume (CV), and tree height (H) also indicate a strong positive correlation, demonstrating that these traits tend to vary in concert across the studied tree individuals. In contrast, crown base height (CBH) is predominantly associated with Dim2 and shows a distinct pattern of variation orthogonal to the main trait cluster, possibly reflecting divergent growth dynamics or adaptive responses to environmental heterogeneity. The color intensity of each data point representing individual trees is calibrated to its cos2 value, a measure of how well the data are represented on the principal components. Data points shaded toward the red end of the spectrum denote a higher cos2 value, indicating a more reliable representation on the PCA plot, while those shaded toward green indicate a lower correspondence. This analysis highlights the major patterns of trait variation and their interrelationships within the population, providing insight into the genetic and environmental factors influencing these morphological traits.

**Figure 5:**
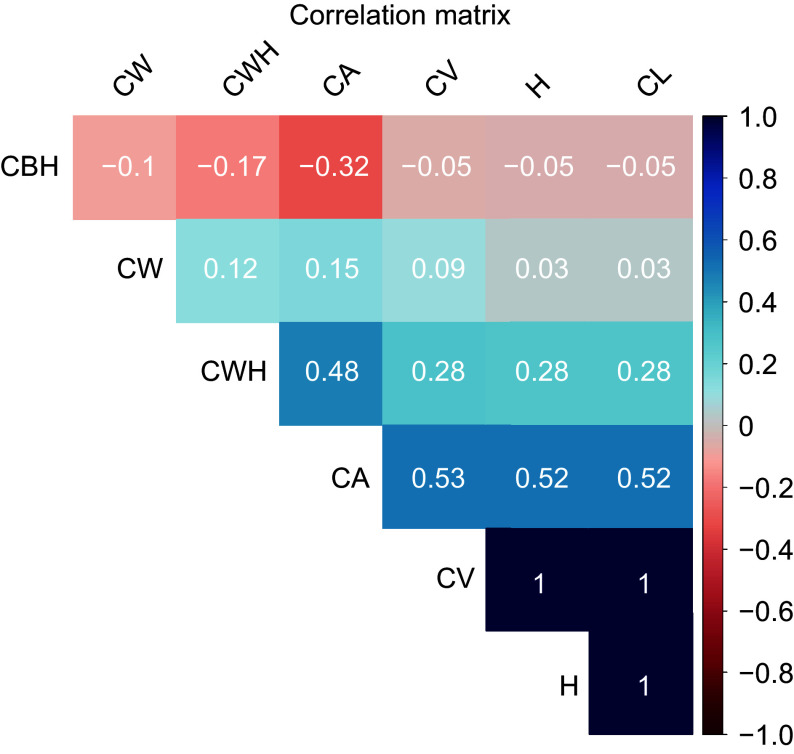
Correlation heatmap of morphological traits in slash pine. The color intensity and sign of the correlation coefficient values range from −1 to +1, where shades of blue represent positive correlations and shades of red indicate negative correlations. A correlation value of 1 implies a perfect positive relationship, −1 indicates a perfect negative relationship, and 0 denotes no correlation.

Figure 5 presents a correlation matrix heatmap showing the pairwise correlation coefficients between different morphological traits of slash pine trees. The color intensity and sign of the correlation coefficient values range from −1 to +1, with blue shades representing positive correlations and red shades representing negative correlations. A correlation value of 1 indicates a perfect positive relationship, −1 indicates a perfect negative relationship, and 0 indicates no correlation. The heatmap provides an immediate visual interpretation of the strength and direction of relationships between traits. Crown Base Height (CBH) shows slightly negative correlations with all other traits, suggesting that as CBH increases, other traits tend to decrease slightly. However, the strength of these relationships is weak, as the values are close to 0. Canopy area (CA) and crown width height (CWH) show a moderate positive correlation, indicated by a lighter blue, meaning that trees with wider crowns generally have greater width at a given height. The strongest positive correlations are observed between canopy area (CA) and canopy volume (CV), CV and tree height (H), and crown length (CL). This means that increases in canopy area are strongly related to increases in crown volume, and similarly, crown volume is closely related to tree height and crown length. These results highlight the interrelated growth patterns of different morphological traits in lodgepole pine and provide insight into their structural and functional relationships.

**Figure 6:**
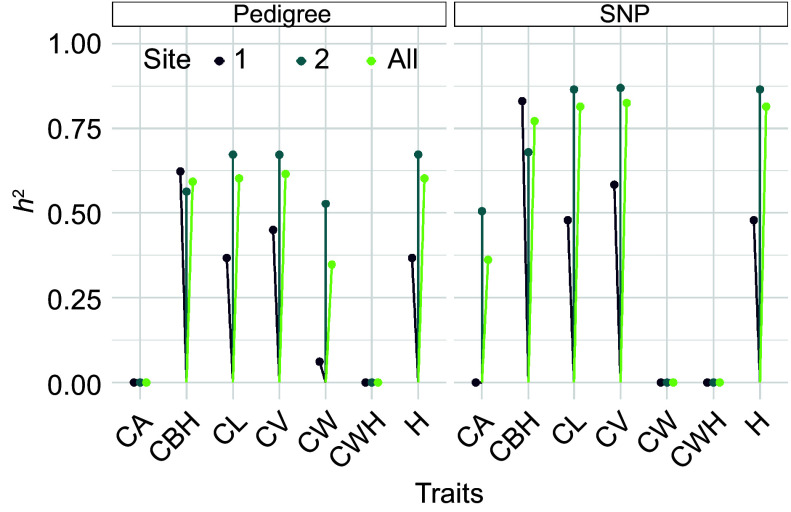
Comparative heritability estimates of slash pine morphological traits using pedigree and SNP data, The vertical lines with colored dots at the end represent the heritability estimates for each trait at different sites. The color of the dots corresponds to different sites, with dark purple indicating Site 1, teal representing Site 2, and yellow green denoting the pooled data across all sites.

Figure 6 illustrates a lollipop plot showing heritability estimates for several morphological traits in slash pine, comparing heritabilities derived from pedigree-based analyses with those obtained from SNP-based approaches. The vertical lines with colored dots at the end represent the heritability estimates for each trait at different locations. The color of the dots corresponds to the different sites, with dark purple representing site 1, teal representing site 2, and yellow-green representing the pooled data across all sites. The left side of the graph, labeled 'Pedigree', shows the heritability estimates for traits such as CA, CBH, CL, CV, CW, CWH, and H as calculated using traditional pedigree information. The right side, labeled 'SNP', shows the heritability estimates for the same set of traits, but derived from SNP marker data. A comparison between the pedigree and SNP approaches shows that the SNP-based estimates tend to show similar or slightly higher heritabilities for traits such as CBH, CL, and CV. This suggests that SNP markers may capture more of the additive genetic variance for these traits. Conversely, for traits such as CW, pedigree-based estimates appear to be more conservative, which may reflect the different efficiency of each method in capturing the underlying genetic architecture. Notably, the heritability for traits such as CBH, CL, CV, and H are relatively high for both methods, indicating that these traits are potentially more influenced by additive genetic factors and could be effectively selected in breeding programs. In contrast, traits such as CWH have lower heritabilities, suggesting a greater influence of environmental factors or non-additive genetic variance. This comparative analysis highlights the strengths and limitations of each approach to estimating heritability, providing valuable insights for slash pine selection and breeding.

**Figure 7:**
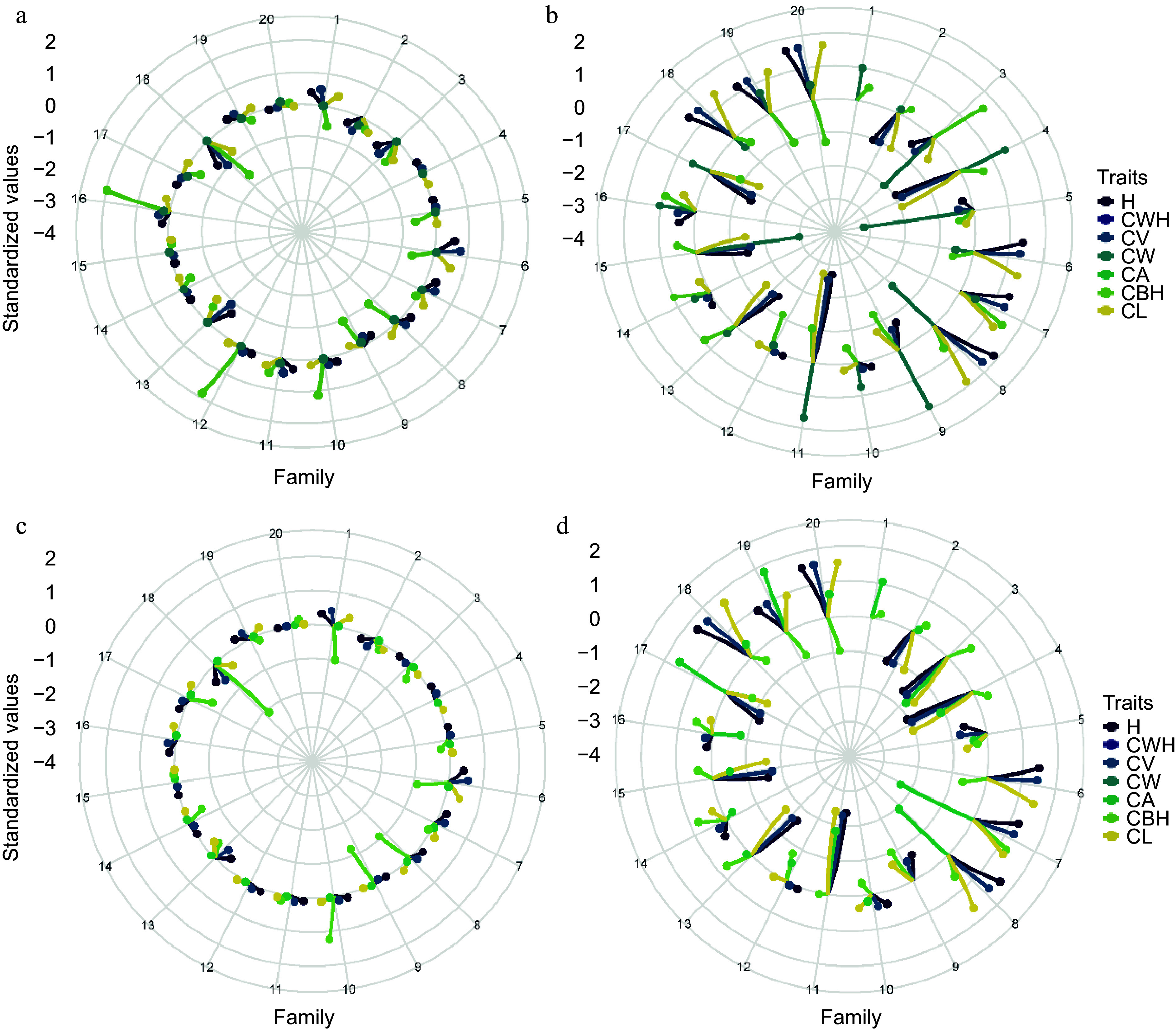
Comparative analysis of pedigree and SNP-based breeding values for morphological traits in slash pine across two sites. (a) Pedigree-based in Site 1, (b) pedigree-based in Site 2, (c) SNP-based in Site 1, (d) SNP-based in Site 2.

Figure 7 consists of four radar plots showing the standardized breeding values of morphological traits for twenty family lines of slash pine. Panels A and C correspond to Site 1, while panels B and D correspond to Site 2. The spokes of the radar charts represent different families, and their radial extent from the center indicates the magnitude of the standardized breeding values for each trait. Panels A and B contrast the pedigree-derived breeding values for Site 1 and Site 2. Families 16 and 12 at Site 1 (Panel A) are notable for their high CBH values, indicating their genetic advantage for this trait. In contrast, at Site 2 (Panel B), families 11 and 9 excel in CW, while families 5 and 15 show negative values for the same trait. Families 18, 19, and 20 at Site 2 show strong breeding values for H, CWH, and CL, indicating their overall genetic superiority for these traits. Panels C and D, which show the SNP-derived breeding values, present a more nuanced picture. At Site 1 (Panel C), there is a general trend toward lower breeding values for most traits, with families 18, 1, 8, and 9 showing negative values for CBH, while family 10 shows a significantly positive value. Conversely, Site 2 (panel D) is more consistent with the pedigree-derived data, with families 18, 19, and 20 maintaining high breeding values. Notably, several families (2, 3, 4, 11, and 15) have negative breeding values for multiple traits, indicating potential genetic limitations. By juxtaposing pedigree and SNP-based analyses, Fig. 7 underscores the complexity of genetic evaluation and the importance of a multifaceted approach to breeding value estimation. The integration of both types of data is critical for a comprehensive understanding of the genetic potential within the breeding program. This comparative analysis highlights the strengths and limitations of each approach and provides valuable insights for the selection and breeding of slash pine.

**Figure 8:**
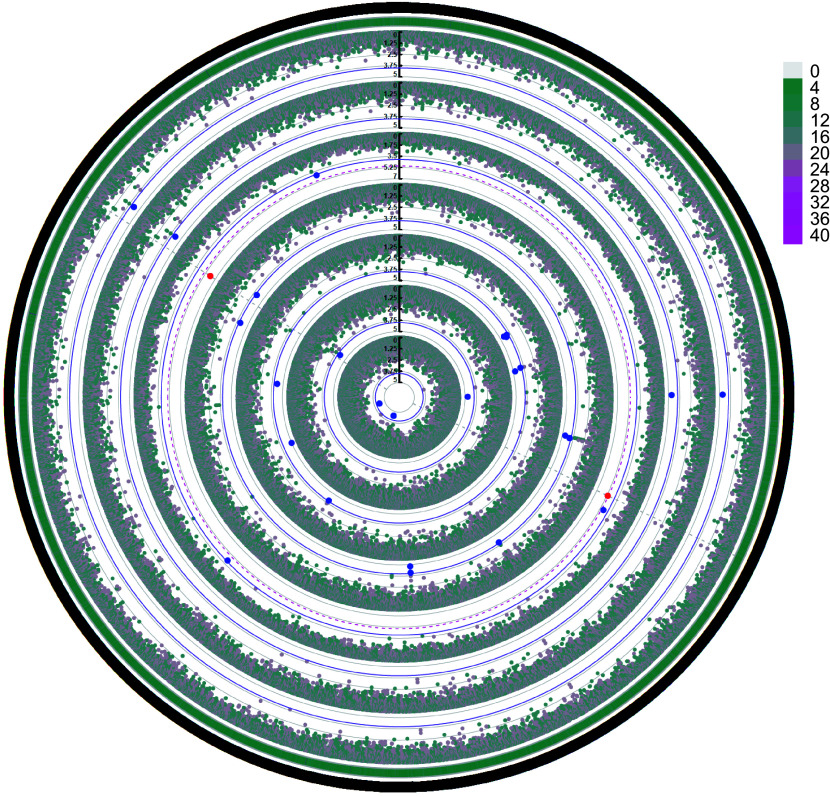
GWAS of the performance of the seven growth traits (from inner to outer: CWH, CV, CW, CA, CBH, CL, H) of slash pine in 2023. The outermost circle represents SNP density, and the grade division is indicated in the legend. Blue dots represent SNP loci reaching a significant association level (α < 0.05), and red dots represent SNP loci reaching a highly significant association level (α < 0.01). The thin purple solid circle lines and red dashed circle lines indicate threshold lines for significant associations with different phenotypes at α < 0.05 and α < 0.01 levels, respectively. The gray solid circle lines correspond to the tick marks of the vertical axis. Only the red significant SNPs identified at the α < 0.01 level are marked by gray dotted lines distributed vertically.

Figure 8 shows a circular Manhattan plot visualizing the results of a genome-wide association study (GWAS) for seven growth traits in slash pine throughout 2023. Each concentric circle in the plot corresponds to one of the traits, ordered from the innermost circle to the outermost as follows: CWH, CV, CW, CA, CBH, CL, and H.

The angular coordinates are mapped to the genomic locations of the SNPs, and the radial distance from the center quantifies the significance level of each SNP association. The outermost circle provides a visual representation of SNP density across the genome, with the significance scale detailed in the legend. Blue and red markers highlight SNPs that reach significant (α < 0.05) and highly significant (α < 0.01) association levels, respectively. The thin purple solid and red dashed circle lines indicate threshold lines for significant associations with different phenotypes at α < 0.05 and α < 0.01 levels, respectively. The gray solid circle lines correspond to the tick marks on the vertical axis. Key SNPs identified for CWH include 'scaffold22007_82420' and 'scaffold44702_108111', suggesting a robust genetic association with this trait. Similarly, for CV, SNPs such as 'scaffold114212_305642' and 'scaffold78970_13765' have been pinpointed as significant. The SNPs associated with CW, such as 'Gene.216560_1274' and 'Gene.216560_1286' indicate important loci for this trait. The SNPs associated with CA and CBH are also mapped accordingly, with SNPs such as 'scaffold124904_157642' and 'scaffold136313_20762' of note. Significant SNPs affecting both CL and H, such as 'scaffold114212_305642' and 'scaffold78970_13765', are found on the outer rings, indicating possible pleiotropic effects or genetic linkage. The plot is highlighted with threshold lines for the α < 0.05 and α < 0.01 levels to help distinguish different levels of significance. The identification of these significant SNPs provides a foundation for further genetic research and potential breeding program improvements in slash pine. This analysis highlights the genetic architecture underlying important growth traits and helps to identify loci that could be targeted for selection in breeding programs.

**Figure 9:**
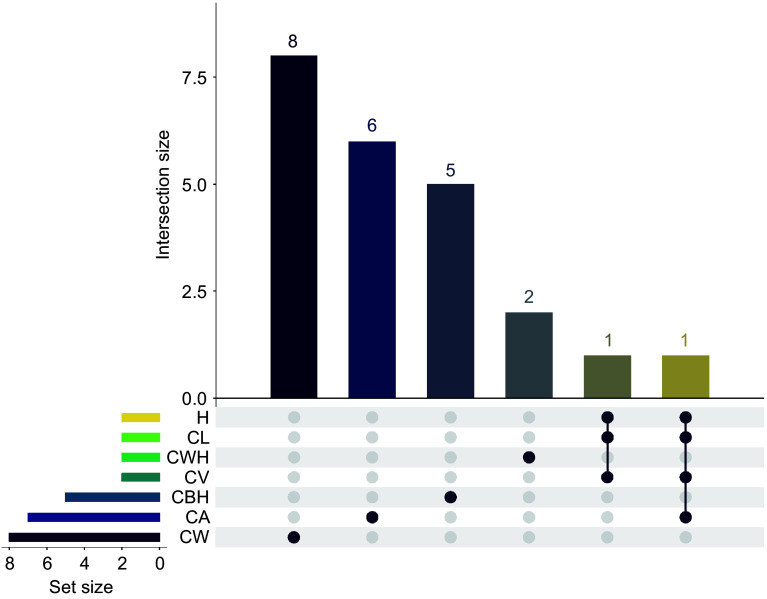
The UpSet plot shows the number of significant GWAS SNP loci for seven traits (CWH, CV, CW, CA, CBH, CL, and H). The matrix plot at the bottom of the UpSet plot shows the intersection conditions between different sets, with each row representing a set and each column representing an intersection combination. The brown dots indicate that the intersection combination includes the set in the corresponding row. The bar graph above the matrix plot shows the number of SNP loci in each trait combination. Set Size refers to the number of SNP loci in each individual group being represented.

Figure 9 presents an UpSet plot showing the overlap of significant genetic loci for seven growth traits in slash pine. The horizontal bars represent the number of significant loci associated with each trait, with CW having the largest number of unique loci (8), followed by CA with 7, and CWH with 2. The vertical bars represent the size of the intersection, which represents the number of loci shared between trait combinations. The plot highlights an intersection between H and CL, where all identified loci are shared, suggesting a potential genetic correlation. In contrast, the CW trait is distinguished by its unique loci, highlighting a specific genetic influence that is distinct from the other traits. The Viridis color scheme enhances the visualization by distinguishing the sets by the number of loci they share. At the base of the matrix, filled circles indicate a trait's participation in an intersection, with lines connecting loci shared by different traits, delineating their commonality. The intersection involving traits H, CL, CA, and CV is particularly highlighted, as indicated by the connected solid circles, indicating a shared genetic basis. In Table 1, a substantial number of genes and SNPs have been identified, each associated with major morphological traits in slash pine. For CWH, two significant SNPs were associated with one gene, PITA_34108. CV was characterized by associations with two genes and two SNPs. CA had a more complex pattern, with seven SNPs associated with six different genes, suggesting a diverse genetic influence. CW was associated with eight SNPs and six genes. For CBH, five SNPs and four genes were identified. Similarly, H and CL were each associated with two genes and two SNPs. These results provide valuable insights into the genetic architecture of important growth traits in slash pine and highlight both unique and common genetic influences that can be targeted for selection in breeding programs.

**Table 1:** Information for 23 canditate genes with 28 significant SNPs that were annotated as growth-related.

Trait	SNP^a^	Gene^b^	Allele^c^	Effect^d^	Gene function
CWH	scaffold22007_82420	PITA_34108	C > T	missenset	Neurofilament heavy polypeptide-like
	scafold44702_108111	−	−	−	−
CV	scaffold114212_305642	PITA_24813	A > T	missense	Ubiquitin family
	scaffold78970_13765	PITA_16597	G >A	downstream_gene	PAR1 protein
CA	scaffold124904_157642	PITA_04981	A >G	synonymous	Fe_2_OG dioxygenase domain-containing protein
	scaffold125333_242817	PITA_17436	A > C	downstream_gene	KH domain
	scaffold165935_108445	PITA_23370	T > C	downstream_gene	−
	scaffold200020_15423	PITA_24554	C > G	missense	Ethylene-responsive element binding factor
	scaffold200020_15465	PITA_24554	G >A	missense	Ethylene-responsive element binding factor
	scaffold71840_181523	−	G > A	intergenic	−
	scaffold78970_13765	PITA_16597	G > A	downstream	PAR1 protein
CW	Gene.216560_1274	Gene.216560	G > A	missense	Putative DEAD-like helicase
	Gene.216560_1286	Gene.216560	A > G	missense	Putative DEAD-like helicase
	Gene.22986_174	Gene.22986	T > C	synonymous	Twinkle homolog protein, chloroplastic/mitochondrial isoform X2
	scaffold10289_45294	PITA_04230	A > C	synonymous	Peptide N-acetyl-beta-D-glucosaminyl asparaginase amidase A
	scaffold103964_286446	−	C > T	intergenic	−
	scaffold230610_125905	PITA_01330	G > A	downstream	Hsp20/alpha crystallin family
	scaffold41862_112441	PITA_00750	G > T	intron	Zinc finger protein CONSTANS-LIKE 6
	scaffold59196_160287	PITA_40818	T > C	intergenic	Arogenate dehydrogenase 2
CBH	scaffold136313_20762	PITA_21834	G > A	missense	Two-component response regulator ORR22
	scaffold139188_163347	PITA_38848	G > C	upstream	Annexin
	scaffold29915_108204	−	T > C	intergenic	−
	scaffold76835_129711	−	A > G	intergenic	−
	super3003_478531	PITA_04633	G > A	downstream	MFS domain-containing protein
H	scaffold114212_305642	PITA_24813	A > C	missense	Ubiquitin family
	scaffold78970_13765	PITA_16597	G > A	downstream	PAR1 protein
CL	scaffold114212_305642	PITA_24813	A > C	missense	Ubiquitin family
	scaffold78970_13765	PITA_16597	G > A	downstream	PAR1 protein
^a^The number after '_' represents the SNP position on the corresponding unigene or scaffold; ^b^depending on the location of the SNP, it is annotated with the corresponding unigene or the gene corresponding to the scaffold; ^c^the first nucleotide is the reference nucleotide, and the second nucleotide is the nucleotide following the substitution event. ^d^the annotation of the SNP, i.e. where the mutation occurred.					

## Discussion

Understanding the nuances of phenotypic trait variations is fundamental in the realm of genetic breeding research. Such insights are instrumental in optimizing the planning and execution of field trials, as well as in the meticulous selection of germplasm resources, thereby elevating the precision and efficacy of the breeding selection process^[39−41]^. In the present study, the application of UAV technology played a pivotal role in accurately capturing spatial phenotypic traits. This approach, having demonstrated its effectiveness in agricultural contexts for growth estimation and yield prediction, is equally valuable in forestry research^[20,42,43]^. The integration of detailed drone-derived phenotypic data with comprehensive GWAS analysis in our slash pine study offers a novel perspective in understanding the genetic underpinnings of these traits.

### Enhanced extraction of tree morphological traits using UAV-derived point cloud data

The extraction of tree morphological traits like CBH, CL, H, CA, CW, and CWH from UAV-derived point cloud data represents a significant leap in forest phenomics. This method, as seen in the present study, harnesses the potential of advanced UAV technology to capture these complex traits accurately. The same study was found in eucalyptus trees^[44]^, where UAV LIDAR and RGB images were used for competitive extraction of morphological structural traits of eucalyptus trees, such as tree height, diameter at breast height (Dbh), crown, trunk, and branches and they demonstrated that these morphological traits obtained based on a 3D point cloud were better than those measured manually. Liu et al.^[45]^ found that point cloud data is outperforms in coniferous tree (Yunnan pine) than other morphological forest species in terms of applicability and accuracy (DBH: Root Mean Square Error (RMSE) = 1.17 cm, Tree Height: RMSE = 0.54 m). Furthermore, the present methodology aligns with the findings of Thiel & Schmullius^[46]^ in terms of the reliability of UAV-derived point clouds, which were shown to be comparable to lidar point clouds. This suggests that the UAV imagery-based point cloud is not only a valid approach but also a potentially more accessible and cost-effective method for extracting tree structural traits. The comparative ease of data acquisition and processing with UAVs could significantly streamline forest phenomics, making it an attractive tool for researchers and forest managers alike.

This side by side proves that 3D point cloud-based morphological features of conifers can be better extracted. In the present study, it was found that many of the under-branch heights were lower than 1.3 m, so the information of the diameter at breast height (DBH) was not obvious, and the DBH metrics were not extracted.

The present approach utilized a series of custom-developed functions, each tailored to measure specific aspects of slash pine morphology. The calculate_CBH_LAS function, for instance, identified the branching point above the trunk using the height thresholds and standard deviation calculations. The calculate_crown_width function and calculate_canopy_height_outside_trunk function provided a comprehensive view of the tree's overall height, mirroring the precision noted in Ghanbari Parmehr & Amati's^[47]^ study on forest canopy traits.

Moreover, the calculate_extreme_points_height function determined the maximum height reached by the canopy, offering insights into the vertical growth extent. The calculate_crown_volume_ellipsoid computed the crown volume, applying an ellipsoidal model to the canopy dimensions derived from the point cloud data. This comprehensive approach to morphological assessment not only highlights the efficiency of UAV technology but also its accuracy, as validated against manually annotated field data, with precision levels exceeding 90% (Supplemental Fig. S1).

The integration of these functions with UAV-derived point cloud data overcomes the limitations of traditional field measurement methods, as noted by Dandois & Ellis^[48]^, who discussed the challenges of manual tree trait measurement in dense forest environments. UAV technology, in contrast, provides a non-invasive, high-throughput, and highly accurate alternative, critical for both growth studies and genetic breeding in trees. While the high accuracy and efficiency of UAV-based methods are clear, challenges remain in data processing and expertise requirements for handling large datasets. Future research should focus on refining data analysis techniques and developing more user-friendly tools to enhance the application of UAV technology in forest phenomics and ecosystem management.

### Genetic variation and GWAS analysis

The present study's heritability estimates and breeding values provide pivotal insights into slash pine's genetic architecture, revealing a spectrum of heritability for various morphological traits. Notably, the findings on tree height heritability surpass those in previous slash pine studies^[49]^. This divergence highlights the comprehensive nature of our phenotypic data and suggests a broader genetic influence on these traits.

The results of this study demonstrate significant disparities in heritability estimates for various morphological traits in slash pine when comparing SNP-based and pedigree-based approaches^[50]^. Specifically, the SNP method exhibits a trend of similar or slightly higher heritability estimates compared to the traditional pedigree approach for traits such as CBH, CL, and CV, suggesting that SNP markers may be more effective in capturing additive genetic variance for these traits, similar results were found by Soleimani et al.^[51]^.

In the context of breeding programs, the relatively high heritability estimates for traits like CBH, CL, CV, and H imply a substantial influence of additive genetic factors^[52]^, making them viable candidates for selection. Conversely, traits with lower heritability, such as CWH, suggest a greater impact of environmental factors or non-additive genetic variance^[53]^.

The breeding value analysis presented in Fig. 7 further underscores the complexity of genetic evaluation. While both pedigree and SNP data highlight genetic advantages for specific families across traits, SNP-derived data introduces nuances, occasionally deviating from pedigree-based results^[54]^, which calls attention to potential genetic limitations. Consequently, a comprehensive understanding of genetic potential within breeding programs necessitates the integration of both pedigree and SNP data^[55]^.

A total of 28 associations were identified by GWAS, including 22 different SNPs localized to 16 different candidate genes. Two of these candidate genes were present at two different mutation sites and were significantly associated with CW and CA traits, respectively, and were annotated as putative DEAD-like helicase and ethylene-responsive ERF, respectively. DEAD-like helicase belongs to the largest subfamily of RNA deconjugating enzymes SF2 and regulates various aspects of plant growth by participating in all biological processes of RNA metabolism in the plant kingdom^[56]^, such as seed development and seedling growth^[57]^, pollen tube formation^[58]^, and post-maturation processing of 23S ribosomes^[59]^. The gene has also been found to play an important role in somatic embryonic processes in Lobelia, where it is not only significantly expressed at the syncytial embryo stage but also induced in response to abiotic stress signals such as JA^[60]^. ERF has also been reported to play an important role in biological processes such as agronomic traits of wheat^[61]^, flower and seed development in Arabidopsis^[62]^, and floral control in maize^[63]^.

Of interest, the mutant locus scaffold114212_305642 was detected in CV, H, and CL traits, respectively. While scaffold78970_13765 was recognized in CV, CA, H, and CL, respectively. The former corresponds to a candidate gene belonging to the ubiquitin family, which modifies the plant proteome to differentiate into appropriate cell and tissue types for maximum adaptation to the environment^[64]^. The latter is annotated as PAR1 protein, which is able to influence photomorphogenesis in plants and can form a network of heterodimeric complexes with the flavonoid sterol-activated HLH transcription factor PRE1 and phytochrome-interacting factor (PIF) to regulate cell elongation and growth^[65]^. The candidate gene significantly associated with CA is also annotated as Fe2OG dioxygenase domain-containing protein, which has been shown to interact with gibberellins and participate in plant growth^[66]^.

The regulation of gene expression at the post-transcriptional level is mainly achieved by proteins that bind RNA recognition motifs (RRMs) and K-homology structural domains (KHs)^[67]^. In this study, the KH domain was recognized in CA, which may be involved in the important transcriptional level regulation during the growth of slash pine. Twinkle homolog protein, chloroplastic/mitochondrial isoform X2 was significantly associated with CW, which is involved in the regulation of maize ectodermal development^[68]^. Peptide N-acetyl-beta-D-glucosaminyl asparaginase amidase A is involved in regulating the activity of macromolecules such as sugars and reducing misfolded proteins in plants^[69]^. Hsp20/alpha crystallin family as an important chaperone protein under heat stress can ensure high productivity of rice^[70]^. The CO (CONSTANS) genes of Arabidopsis, rice and barley, which play an important role in the regulation of flowering by photoperiod^[71]^, were also identified as CW significantly related genes. For CBH trait, three candidate genes were identified, namely Two-component response regulator ORR22, Annexin, and MFS domain-containing protein. Among them, Annexin binds to membrane phospholipids in a calcium-dependent manner and is widely involved in the regulation of plant growth and MFS proteins are the largest group of secondary membrane transporter proteins in the cell, and can function as transporter proteins with a variety of substrates in plant growth and development^[72]^. These associations highlight the link between significant SNPs and known functional genes or biological pathways, providing an intuitive understanding of their biological significance. Consequently, the above results indicate that the GWAS in this study has effectively revealed the genetic basis of growth trait variations in slash pine.

## Limitations and future directions

In our study on slash pine morphological traits and genetic breeding, several limitations and future research directions merit consideration.

Firstly, the sample size, while substantial, may not fully represent the genetic diversity within slash pine populations. Expanding the sample size and including individuals from diverse geographical regions could enhance the robustness of the present genetic findings.

Secondly, environmental factors such as soil composition, climate, and inter-tree competition are known to influence tree growth. Integrating environmental data into the analyses would enable a more comprehensive understanding of gene-environment interactions.

Furthermore, while the GWAS analysis identified candidate genes associated with morphological traits, further functional studies and validation experiments are needed to confirm the roles of these genes in slash pine growth and development.

Lastly, future research directions may involve the integration of multi-omics data, including transcriptomics and proteomics, to unravel the intricate regulatory networks involved in tree growth and morphology.

## Conclusions

In summary, the present study delves into the realm of slash pine genetics and morphology, shedding light on key aspects of phenotypic variation and genetic breeding. Through the innovative use of UAV technology, efficient and highly accurate measurements of critical morphological traits were achieved, revolutionizing the way we collect data in forestry research. The present results underscore the importance of morphological traits, such as tree height and crown dimensions, in understanding growth patterns and structural variation in slash pine. UAV technology has proven to be a game changer, providing rapid and accurate data collection compared to traditional labor-intensive methods. In addition, the integration of detailed drone-derived phenotypic data with comprehensive GWAS provided a new perspective on the genetic basis of these traits. The identification of candidate genes associated with specific morphological traits paves the way for more precise genetic improvement and breeding strategies in slash pine.

In addition, the practical implications of the present findings for forest management and breeding programs are profound. Forest inventory processes and monitoring efforts can be greatly enhanced by the ability to quickly and accurately assess morphological traits using UAV technology. This technological advance will allow foresters to make more informed decisions regarding forest health, growth patterns, and resource allocation. In addition, the identification of candidate genes associated with key morphological traits provides valuable markers for selective breeding programs aimed at improving growth rates, disease resistance, and overall productivity of slash pine. Implementation of these genetic findings in breeding programs may lead to the development of superior slash pine cultivars that are better able to withstand environmental stresses and meet the demands of sustainable forestry practices.

While the present study represents a significant leap forward, it is important to acknowledge the limitations, such as sample size constraints and the need for further validation of candidate genes. These limitations guide us toward future research directions that include expanding sample diversity, refining data processing methods, considering environmental factors, and integrating multi-omics data for a more comprehensive understanding.

## SUPPLEMENTARY DATA

Supplementary data to this article can be found online.

## Data Availability

The data mentioned in this paper are available upon request from the corresponding author. The codes and examples of point cloud data used in this study have been backed up to a cloud drive, which can be accessed at: https://pan.quark.cn/s/1dbde2d1f7fc.
