# Supplementary figures and images for: Enhancing genomic association studies in slash pine through close-range UAV-based morphological phenotyping
Source: For Res (Fayettev). 2024 Jul 25;4:e025. doi: 10.48130/forres-0024-0022 (PMC11524239; doi:10.48130/forres-0024-0022)

H

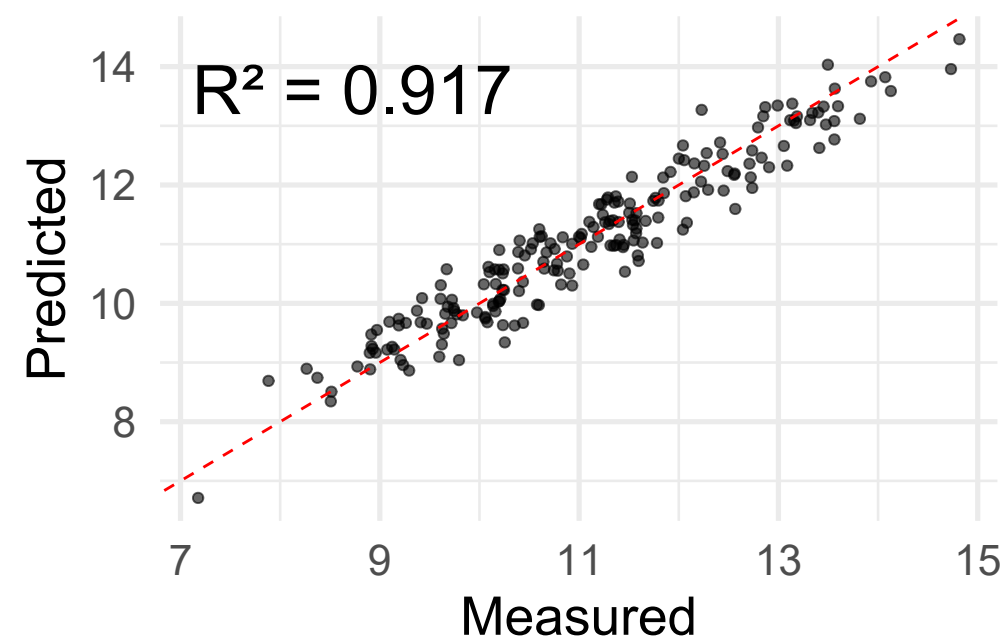

CWH

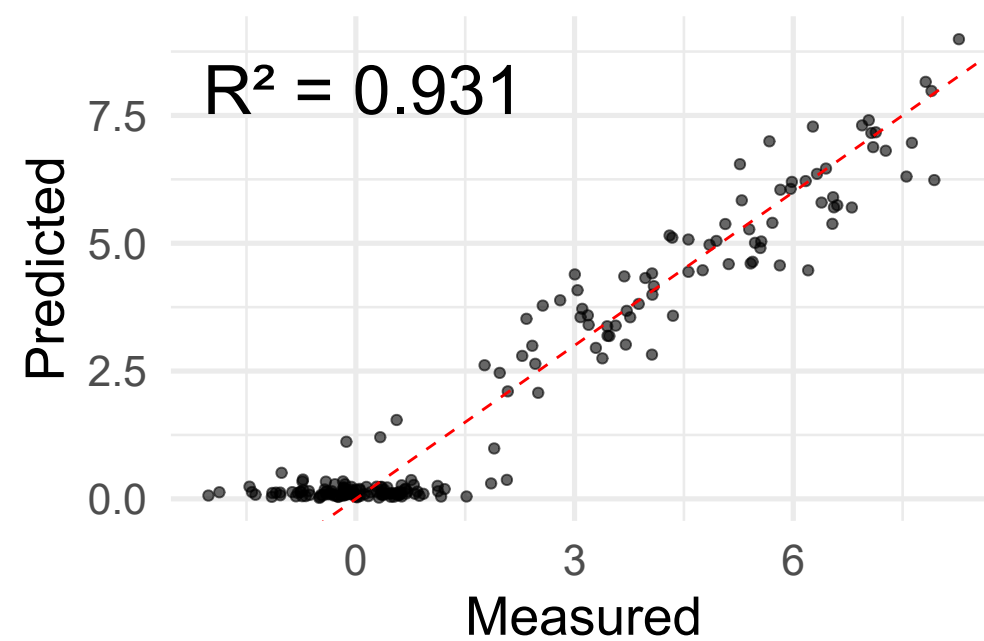

CV

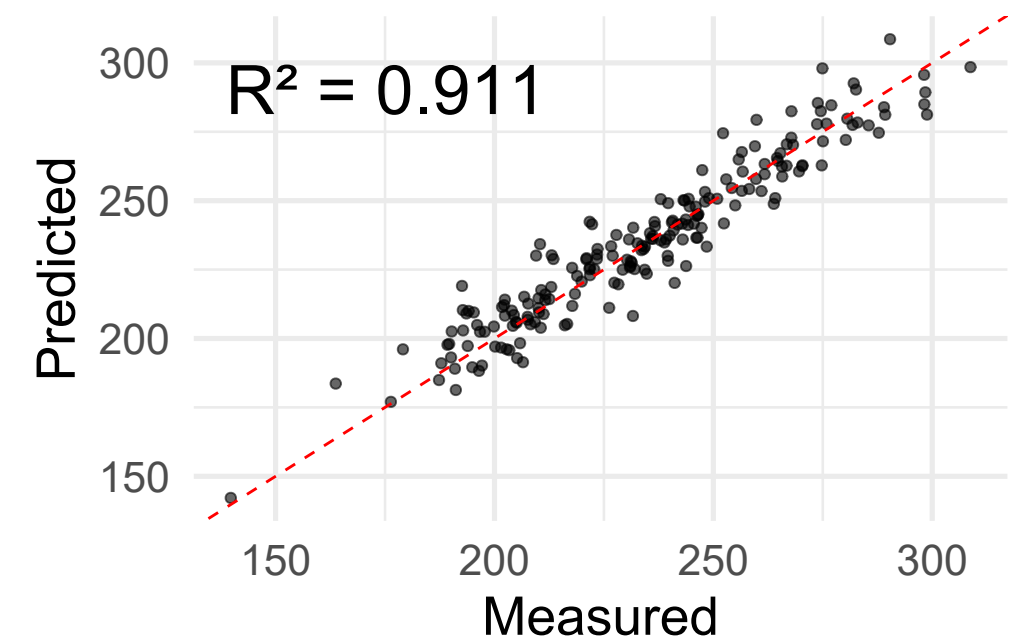

CW

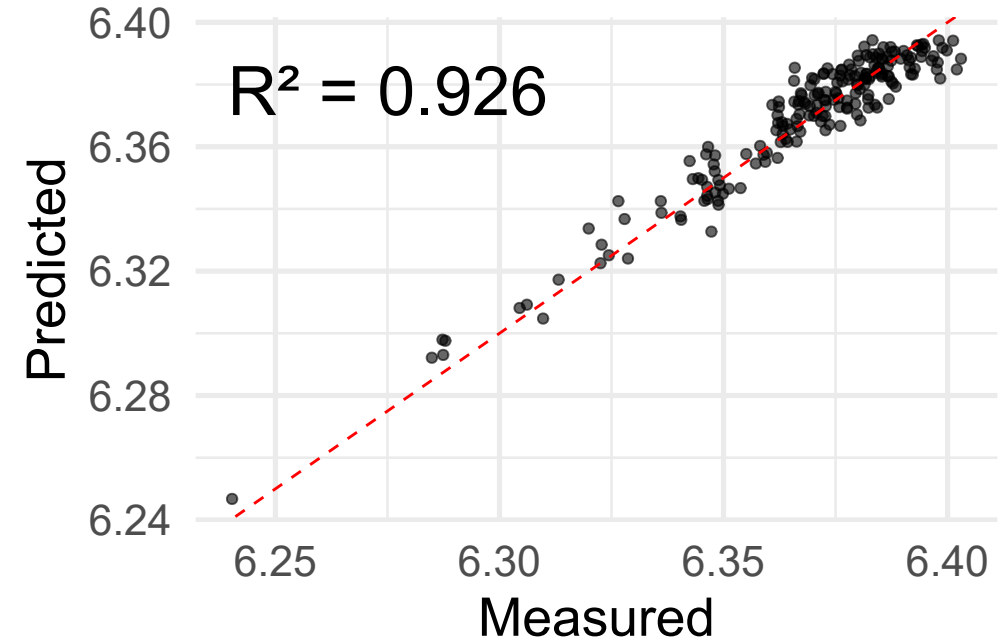

CA

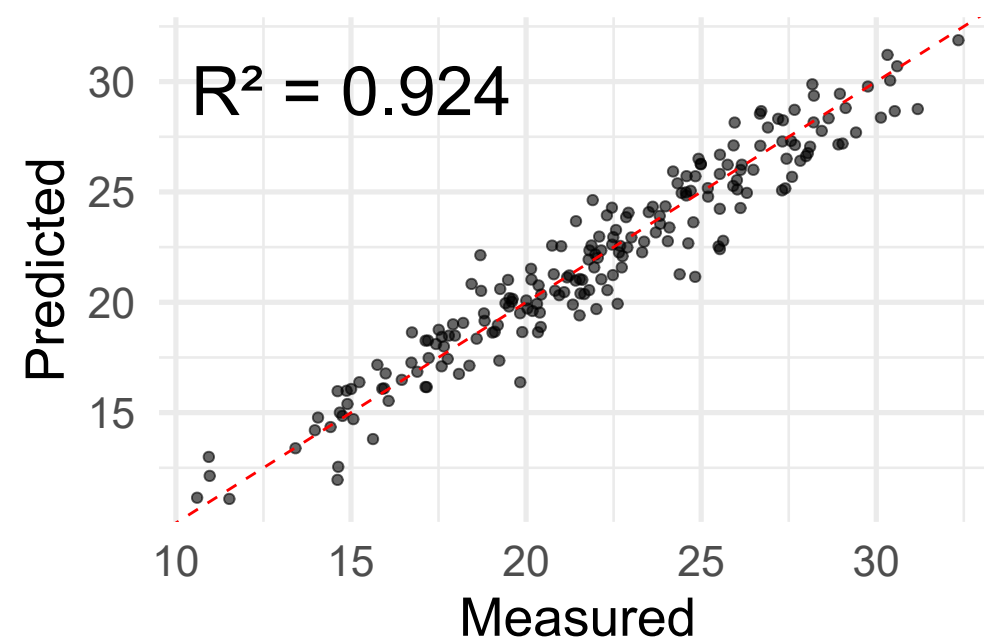

CBH

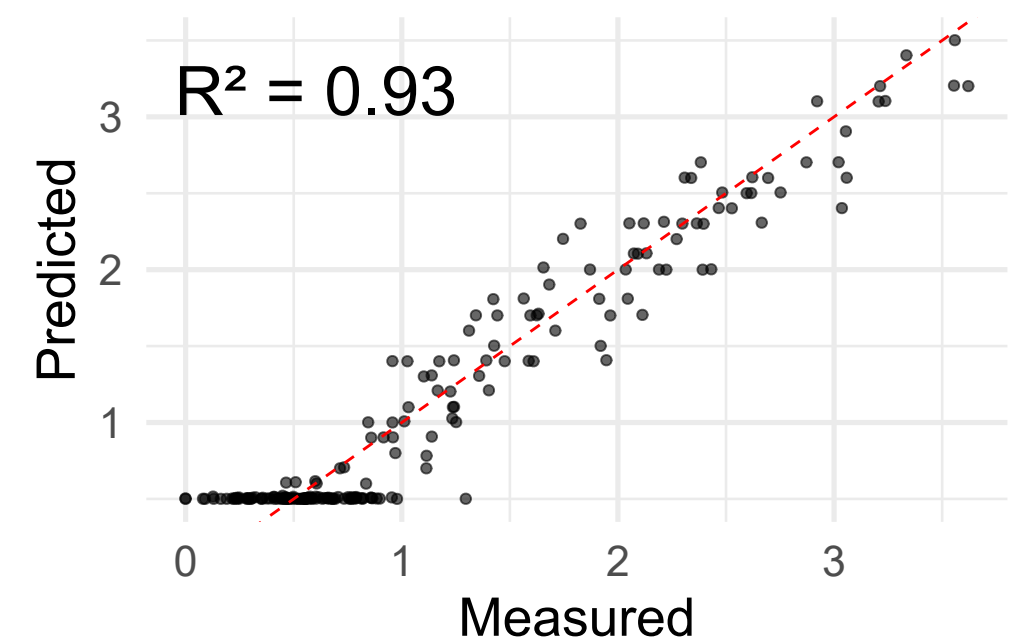

CL

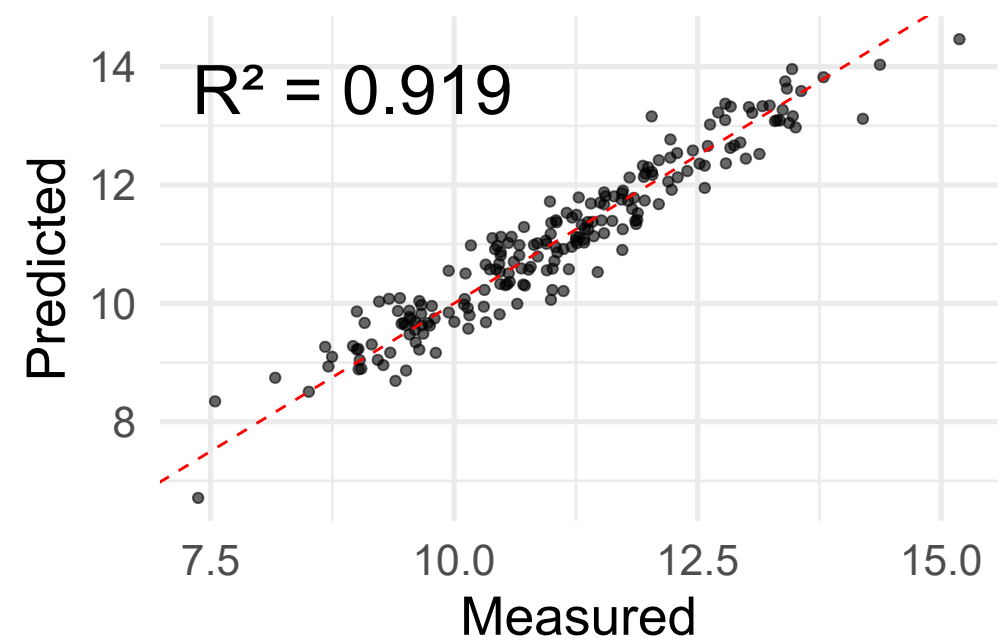

Supplement: Supplementary file 1 — Supplementary data to this article can be found online. [file forres-0024-0022-S1.pdf]
